# Treatment preference among patients with spinal muscular atrophy (SMA): a discrete choice experiment

**DOI:** 10.1186/s13023-020-01667-3

**Published:** 2021-01-20

**Authors:** Alisha Monnette, Er Chen, Dongzhe Hong, Alessandra Bazzano, Stacy Dixon, W. David Arnold, Lizheng Shi

**Affiliations:** 1 Department of Health Policy and Management, Tulane University School of Public Health and Tropical Medicine, 1440 Canal Street, Suite 1900, New Orleans, LA 70112 USA; 2 Department of Global Community Health and Behavioral Sciences, Tulane University School of Public Health and Tropical Medicine, New Orleans, LA USA; 3 Genentech Inc., San Francisco, CA USA; 4 Department of Neurology, University of Colorado School of Medicine, Aurora, CO USA; 5 Department of Neurology, The Ohio State University, Columbus, OH USA

**Keywords:** Spinal muscular atrophy, Discrete choice experiment, Patient preferences, Treatment attributes, Nusinersen, Onasemnogene abeparvovec-xioi

## Abstract

**Objective:**

To examine patient/caregiver preference for key attributes of treatments for spinal muscular atrophy (SMA).

**Background:**

In the rapidly evolving SMA treatment landscape, it is critically important to understand how attributes of potential treatments may impact patient/caregiver choices.

**Design/methods:**

A discrete choice experiment survey was developed based on qualitative interviews. Patients with SMA (≥ 18 years) and caregivers of patients were recruited through a U.S. patient organization. Respondents made choices in each of 12 sets of hypothetical treatments. The relative importance of five treatment characteristics was compared (measured by regression coefficients [RC] of conditional logit models): (1) improvement or stabilization of motor function, (2) improvement or stabilization of breathing function, (3) indication for all ages or pediatric patients only, (4) route of administration [repeated intrathecal (IT) injections, one-time intravenous (IV) infusion, daily oral delivery] and (5) potential harm (mild, moderate, serious/life threatening).

**Results:**

Patient ages ranged from less than 1 to 67 years (n = 101, 65 self-reported and 36 caregiver-reported) and 64 were female. Total SMA subtypes included: type 1 (n = 21), type 2 (n = 48), type 3 (n = 29), other (n = 3). Prior spinal surgery was reported in 47 patients. Nusinersen and onasemnogene abeparvovec-xioi use were reported in 59 and 10 patients, respectively. Improvement in motor and breathing function was highly valued [RC: 0.65, 95% confidence interval (CI): 0.47–0.83 and RC: 0.79, 95% CI: 0.60–0.98, respectively]. Oral medication and one-time infusion were strongly preferred over repeated IT injections (RC: 0.80, 95% CI: 0.60–0.98 and RC: 0.51, 95% CI: 0.30–0.73, respectively). Patients least preferred an age-restricted label/approved use (≤ 2 years of age) (RC: − 1.28, 95% CI: − 1.47 to − 1.09). Cross-attributes trade-off decision suggested a lower willingness for a high-risk therapy despite additional efficacy gain. For some patients, there may be willingness to trade off additional gains in efficacy for a change in route of administration from repeated intrathecal administration to oral medication.

**Conclusions:**

Improvements in motor/breathing function, broad indication, oral or one-time infusion, and minimal risk were preferred treatment attributes. Treatment decisions should be made in clinical context and be tailored to patient needs.

## Introduction

Spinal muscular atrophy (SMA) is a rare and debilitating genetic neuromuscular disease caused by a loss of function mutation or deletion of the survival motor neuron gene 1 (SMN1) [1–3]. It affects one in approximately 15,000 live births [4, 5]. SMA is characterized by progressive loss of motor neurons, muscle weakness, and atrophy [6]. There are five primary types of SMA numbered from 0 (most severe) to 4 (least severe), based on age of onset and highest physical milestone achieved. The disease is a continuum from most severe to the milder phenotype form. Patients with type 0, a very rare and the most severe form of SMA, have onset of symptoms at birth and usually die beyond 6 months of age. Type 1 SMA is the most common form of SMA with onset of symptoms between 0 and 6 months. Patients with type 1 SMA present a number of functional impairments, such as severe hypotonia, progressive loss of motor function, impaired swallowing and respiratory function, and cannot sit independently during their reduced lifespan. Patients with type 2 or type 3 SMA experience disease onset before and after the age of 18 months, respectively. Those with type 2 SMA are unable to stand or walk without support, in contrast to patients with type 3 SMA who are able to stand and walk until the disease progresses. Type 4 SMA onset is in adulthood and that causes milder muscle weakness [7].

SMA is associated with direct and indirect costs of approximately $1 billion annually in the United States [8, 9]. Given the rarity of SMA, the direct and indirect costs per capita are close to $200,000 [10, 11]. The latest treatment patterns and economic study on patients with SMA found significantly higher healthcare utilization and costs than the general population [12]. Multiple studies have found substantially higher costs for patients with SMA compared to healthy populations [10, 11, 13]. In addition to impairment on quality of life and social participation among patients with SMA, SMA was associated with reduced caregiver quality of life, which was related to patient burden, impaired psychosocial well-being, and loss of caregiver’s productivity [14–16].

Despite the progress in SMA treatment with three important landmark treatments (approved by the Food and Drug Administration [FDA]), the disease burden and related unmet need remains significant for many patients with SMA and their caregivers [5, 17]. Spinraza® (nusinersen) is a disease-modifying treatment approved for all patients with SMA and is administered via intrathecal (IT) injection every 4 months after a series of loading doses [18]. While repeated intrathecal injections are the standard approach to achieve adequate intrathecal distribution and targeting of the central nervous system (CNS), alternatives such as intrathecal catheter systems are being explored [19, 20]. Nusinersen is a survival motor neuron-2 (SMN2) directed antisense oligonucleotide that enhances production of full-length survival motor neuron (SMN) protein and slows the progression of the disease. Zolgensma® (onasemnogene abeparvovec-xioi) is a gene replacement therapy that uses a viral vector to deliver a functional copy of the *SMN1* gene. Onasemnogene abeparvovec-xioi is administered as a one-time intravenous (IV) infusion and is only approved for the treatment of children less than 2 years of age because of current limitations of dosing (i.e. viral titers and increased likelihood of immune response) and the fact that this drug has only been tested for this age group [17, 21, 22]. The most recent treatment approved by the FDA is Evrysdi® (risdiplam), an orally administered, SMN2 splicing modifier for patients 2 months of age and older with SMA [23, 24]. The drug increases exon 7 inclusion and thus full-length SMN protein production from the SMN2 gene [23]. The SMA treatment landscape continues to evolve as there are several other medications in development.

The unique health status and life experience of each patient and family affected by SMA may influence how they perceive meaningful changes with respect to desired benefits or avoided risks from a therapeutic agent. With multiple treatments available, both healthcare professionals, as well as patients and caregivers, have many considerations to consider when deciding on the right treatment choices [25–28]. A 2007 consensus statement, issued by the International Standard of Care Committee for SMA, to address the 5 priority care areas for SMA, found that for those affected by type 1 SMA, high priority components of meaningful change relate to immediate concerns with breathing, feeding, swallowing, and the ability to communicate [29]. For patients with types 2 and 3 SMA, priorities include disease stabilization, independence and the ability to perform basic personal tasks, and reducing muscle fatigue [29]. In most cases, patients and caregivers perceive benefit in maintenance of current abilities and avoiding decline in function. They indicate that even small improvements in functional abilities would be meaningful, especially if these contribute to the increase in ability to independently perform activities of daily living [29].

Despite the burden of SMA, little focus has been given to understand how patients and caregivers weigh the possible risks associated with therapies against potential benefits associated with those therapies [28]. Patients’ perceptions of benefit-risk are essential to informing the drug approval process and the context in which potential therapies are evaluated. In light of three current SMA treatments and more treatment options on the horizon, a unique window of opportunity arises to assess and characterize preferences for patients and their caregivers on SMA treatment characteristics and attributes. Therefore, the primary objectives of this study were to quantify benefit-risk trade-off preferences and to estimate the relative importance of hypothetical treatment attributes. This study addresses a significant gap in the evidence base for SMA, particularly given patient and caregiver perceptions of benefit-risk tradeoffs that inform the patient-centered value framework for the innovative therapies. Further, this study has the ability to provide evidence to support the need for informed joint treatment decision making for SMA patients by educating providers to understand patient and family preferences, and informing payers about the unique role each therapy may bring; thus, allowing equitable benefit design so patients have access to treatments that work best for them.

## Methods

### Study design

We designed and administered a discrete choice experiment (DCE) survey with input from SMA specialists and CureSMA, a U.S organization that provides support to patients and families affected by SMA and funds and directs research for SMA. Specifically_,_ the study asked for patient/caregiver’s preferences for treatment characteristics such as route of administration, mechanism of action, safety, efficacy, treatment schedule, clinical data availability, and out-of-pocket costs. The theoretical background and rationale supporting DCEs have been discussed in detail by Johnson et al. in a report that summarizes good research practices for constructing DCEs [30].

**Fig. 1:**
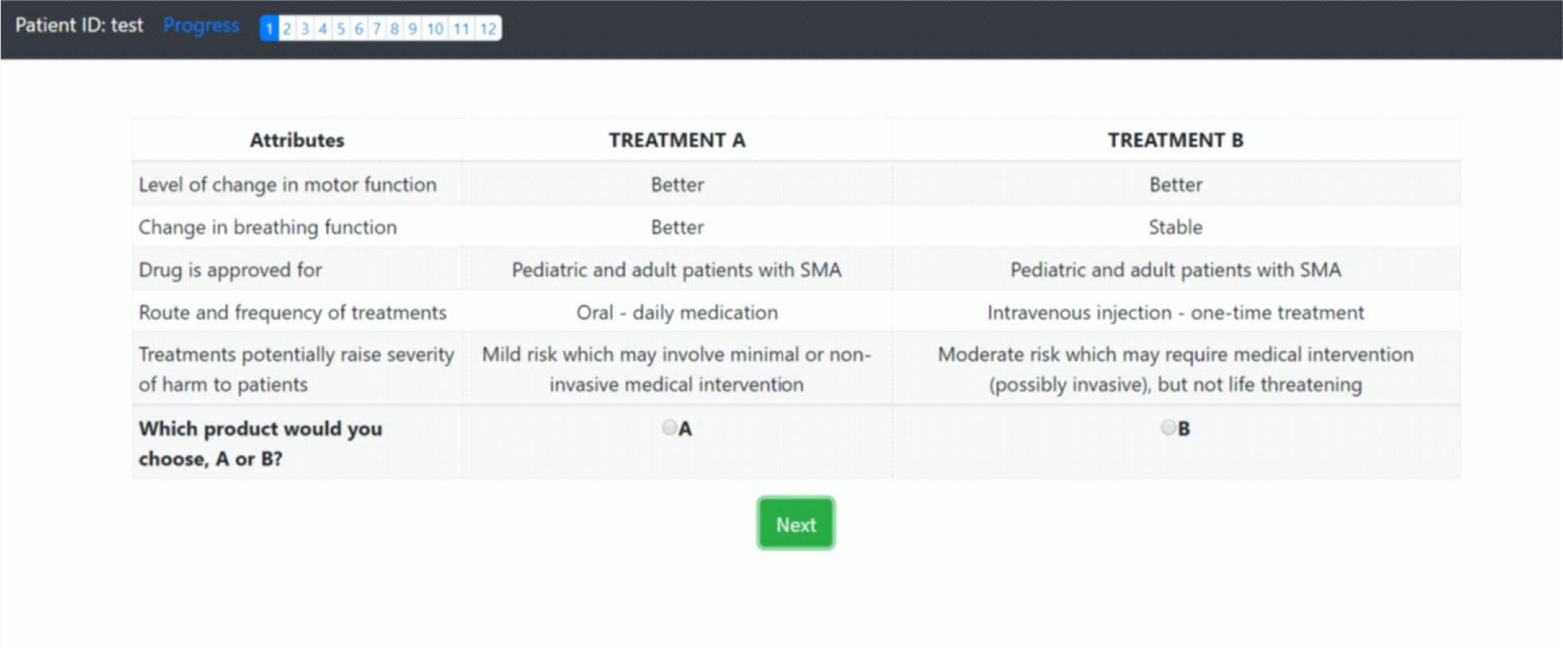
Snapshot of SMA survey instrument detailing treatment and attribute choices

In a DCE, respondents must choose their most preferred treatment alternative from two hypothetical treatment profiles, assuming that these are the only treatment options available. DCEs therefore allow one to compare the relative importance of various treatment attributes. The DCE survey instrument requires constructing a series of 8 to 12 trade-off questions that evaluate SMA treatments for patients. Each hypothetical treatment profile consists of combinations of attribute levels. Figure 1 displays a screenshot of the actual survey instrument. The image shows an example of how a question is displayed. In general, the survey questionnaire should be completed within 20–25 min. The combination of attributes and levels that respondents evaluate in a choice-experiment survey is known as the experimental design. These combinations must have statistical properties that allow estimating the preference weights of interest.

### Study population

Patients (aged ≥ 18 years) and caregivers of SMA patients with SMA types 1–4 were recruited through CureSMA. Respondents were included in the study if they were able to read and understand English, to provide informed consent and complete the survey instrument, and willing to participate in the study. The study was approved by Tulane University Institutional Review Board (Study Number: 2018-2197).

### Survey instrument development

Prior to developing the survey instrument, a comprehensive literature review was conducted to identify important treatment characteristics (also known as attributes) in SMA. The attributes chosen for inclusion in this study had to meet three criteria: (1) clinically relevant and address potential concerns of physicians and other decision makers who make treatment recommendations or reimbursement decisions (i.e. content validity), (2) meaningful and salient to study respondents (i.e. face validity), and (3) supported by evidence as through literature reviews, focus groups, or other scientific methods [31, 32].

The number of attributes that can be accommodated in a choice-experiment survey instrument depends on the complexity of the trade-off task, the degree of familiarity the respondents have with the attributes included in the trade-off tasks, respondent attentiveness in answering survey questions, and the number of respondents participating in the survey. Task difficulty increases with the number of attributes and the number of attributes that are probabilistic. Measurement error increases with task difficulty, which decreases the precision with which preference weights can be estimated for any given sample size [31, 32]. Therefore, the number of attributes included in the final survey instrument were limited to those that are necessary to answer the research question and that meet the criteria outlined above.

A choice-experiment study design with five attributes, each with two to five levels, and eight to ten choice questions per respondent requires approximately 100 respondents to estimate a preference model with acceptable precision for all parameters [31]. Since the DCE study, for which this instrument is designed, aims to enroll 100 subjects, five attributes in the final survey instrument were needed. Thus, following the literature review, eight attributes were subject to pretesting through qualitative interviews conducted on three patients and one caregiver as well as two specialty providers for SMA to validate the treatment characteristics and narrow down the attributes to five. In addition to the final five attributes included in the survey, we also captured clinical information and demographics about the respondents, both for patients and caregivers, such as age, sex, race/ethnicity, employment status, income level, insurance status, education level, marital status, SMA treatment history and status, and clinical history including past surgeries.

Once the survey instrument was developed, we conducted one-on-one, semi-structured cognitive interviews with a convenience sample of respondents (i.e., patients with SMA and caregivers). Cognitive interviews were done to test the understandability of the survey instrument, the appropriateness of descriptive information, and the cognitive burden of the trade-off questions. We conducted pilot testing by interviewing five respondents with SMA. All interviews were conducted via teleconference or video-conference.

During the pretest interview, respondents were asked to complete the draft survey in field condition, or if the respondent preferred, the interview was completed while the researcher (AB) followed along on-screen during the completion of the interview. After completing the survey in this manner, respondents were asked a series of debriefing questions to determine whether they understood the definitions and instructions, accepted the hypothetical context of the survey, and successfully completed the choice questions in the survey instrument as instructed. All pretest interviews were conducted by a research member (AB) who had extensive experience interviewing survey respondents; and a second researcher observed to ensure quality control. The pretest interviews ensured that all changes to the survey instrument were working as expected and assessed how long it would take respondents to complete the survey under field conditions. Using the findings from the pretest interviews, the survey was finalized and programmed on a web-based survey platform, REDCAP [33]. Table 1 displays the treatment attributes and levels.

**Table 1:** Treatment attributes and levels

Category	Attribute	Levels	Code
Motor function	Level of change in motor function	Stable	1
		Better	2
Breathing function	Level of change in breathing function	Stable	1
		Better	2
Indication	Drug is approved for	Pediatric and adult patients with SMA	1
		Pediatric patients with SMA only (e.g., < 2 years of age)	2
Route and frequency of administration	Route and frequency of treatments	Intravenous (IV) infusion—one-time treatment	1
		Intrathecal (IT) spinal injection—3–6 times per year	2
		Oral—daily medication	3
Potential harm	Treatments potentially raise severity of harm to patients	Mild risk which may involve minimal or non-invasive medical intervention	1
		Moderate risk which may require medical intervention (possibly invasive), but not life threatening	2
		Serious, or life-threatening risk which may require urgent medical intervention or prolonged hospitalization	3

The web-based survey instrument was administered remotely through an email-invitation to up to 100 respondents. Patients and caregivers were enrolled by consent through electronically signing an informed consent and an incentive, valued at $50.00 USD, was issued (via mail or e-mail) to compensate for their time.

### Sample size

Sample size was estimated based on the main effects in our statistical model by Louviere’s sample size estimation method [34]. Sample-size calculations represent a challenge in the choice experiment [35]. The minimum sample size depends on a number of criteria including the question format, the complexity of the choice task, and the desired precision of the results [34]. Based on the literature, a choice-experiment study design with 5 attributes, each with 2–3 levels, and 8 to 12 choice questions per respondent requires approximately 50–100 respondents to estimate a preference model with acceptable precision for all parameters [36]. Therefore, we aimed to recruit 80 patients and 20 caregivers, based on the suggestions from SMA specialists and CureSMA, as majority of people that are registered and actively participate within the CureSMA network are adult patients. This sample size was deemed sufficient to estimate the main effects in our statistical model by Johnson and Orme method [35].

### Discrete choice experiment/statistical analysis

Based on the treatment attributes, each respondent received a series of 12 trade-off questions that evaluate SMA treatments for patients and caregivers. A D-optimal algorithm was used to construct a fractional factorial experimental design [37]. A total of 12 pairs in the experimental design were generated to estimate both main effects and possible selected interaction effects. Main effects included the preference weight for each attribute level independent of the other attributes and levels included in the study. Interaction effects tested whether the effect of one attribute level was independent of the other attributes and levels in the study. Descriptive statistics were performed on demographic and clinical characteristics.

To investigate preferences for SMA therapies, a theoretical framework based on a conditional logit model was employed to allow attribute coefficients to vary across respondents, accounting for preference heterogeneity and improving the realism of model assumptions [38]. Conditional logit models adjusted the standard errors of utility estimates to account for repeated choices by the same individual.

The results of this analysis include estimating log-odds relative preference weights and odds-ratio tests. All estimates were reported with 95% confidence intervals. Additionally, we controlled for the effect on preference estimates of respondent-specific health history and current disease conditions as well as other individual characteristics. These results allowed us to calculate: (1) predicted choice shares for two treatments with specified attribute levels, indicating the predicted proportion of respondents who would choose each treatment profile, and (2) relative importance of attributes over the ranges of attribute levels included in the DCE.

## Results

There were 101 respondents (65 self-reported patients and 36 caregivers) included in the analysis. Table 2 displays the clinical and demographic characteristics of both patients and caregivers. Patient ages ranged from less than 1 year old to 67 years old, 64% were female, and 81% were white. The household income across respondents varied with 21% having less than $25,000 and 22% of respondents having $100,000 or more.

**Table 2:** Demographic characteristics for SMA patients and caregivers (N = 101)

	Patient (n = 65)	Patient represented by caregiver (n = 36)	Caregiver (n = 36)
Age, years (SD)	36 (12.1)	10 (12.2)	38 (10.9)
Age range, years	18 to 67	0.2 to 42	23 to 69
**Gender, n (%)**			
Male	18 (27.7)	17 (47.2)	
Female	46 (70.8)	19 (52.8)	
Prefer not to answer	01 (01.5)		
**Race, n (%)**			
White	56 (86.2)	26 (72.2)	
Black	02 (03.1)	04 (11.1)	
Asian	06 (09.2)	01 (02.8)	
Don't know/prefer not to answer	01 (01.5)	05 (13.9)	
**Hispanic or Latino, n (%)**			
Yes	03 (04.6)	13 (36.1)	
No	60 (92.3)	22 (61.1)	
Don't know/prefer not to answer	02 (03.1)	01 (02.8)	
**Marital Status, n (%)**			
Single	40 (61.5)		07 (19.4)
Married	22 (33.8)		28 (77.8)
Separated/divorced/widowed	03 (04.6)		01 (02.8)
**Family income in last 12 months, n (%)**			
$100,000 or more	16 (24.6)		06 (16.7)
$75,000 to $99,999	05 (07.7)		05 (13.9)
$50,000 to $74,999	11 (16.9)		11 (30.6)
$25,000 to $49,999	09 (13.8)		06 (16.6)
Less than $25,000	14 (21.5)		07 (19.4)
Don't know/prefer not to answer	10 (15.4)		01 (02.8)

Table 3 presents the clinical and disease characteristics for SMA patients. In total, there were 21 patients with type 1 SMA, 48 patients with type 2 SMA, 29 patients with type 3 SMA, and 1 patient with type 4. A majority of patients (68%) had their first symptoms before they were 18 months old. 58% of patients were on nusinersen at the time of survey completion. Almost half of the study respondents reported motor function without assistance (42%) and 84% reported breathing function without mechanical support.

**Table 3:** Clinical characteristics for SMA patients (N = 101)

	Patient (n = 65)	Patient represented by caregiver (n = 36)
Age range when first diagnosed with SMA	6 mos. to 59 yrs	prenatal to 26 yrs
**Age when first symptoms associated with SMA, n (%)**		
< 6 months old	13 (20.0)	18 (50.0)
6–18 months old	30 (46.2)	11 (30.5)
18 months—3 years old	13 (20.0)	03 (08.3)
4–18 years old	07 (10.8)	01 (02.7)
> 18 years old	02 (03.1)	02 (05.5)
**Type of SMA, n (%)**		
Type 1	04 (6.15)	17 (47.22)
Type 2	33 (50.77)	15 (41.67)
Type 3	27 (41.54)	02 (05.55)
Type 4	01 (1.54)	0
Unknown	0	02 (05.55)
**Self-reported level of physical (motor) functioning or ability, n (%)**		
Walking alone	8 (12.31)	1 (2.77)
Sitting without support	23 (35.38)	10 (27.78)
Walking with assistance	7 (10.77)	3 (8.33)
Standing with assistance	1 (1.54)	1 (2.77)
Hands and knee crawling	0	2 (5.55)
No motor function	3 (4.62)	3 (8.33)
None of the above; have some motor function	23 (35.38)	16 (44.44)
**Self-reported breathing ability, n (%)**		
Can breathe without mechanical support	55 (84.61)	29 (80.55)
Need mechanical support to breathe for some of the day	7 (10.77)	3 (8.33)
Need mechanical support to breathe for most of the day	1 (1.54)	0
Need 24-h mechanical support to breathe	2 (3.08)	4 (11.11)
**Other illness limits daily activities, n (%)**		
Yes	10 (15.38)	2 (5.55)
**Current Treatment, n (%**)^a^		
Supportive care only (not currently using a medical treatment for SMA)	14 (21.54)	6 (16.67)
Spinraza® (Nusinersen)	41 (63.08)	18 (50.00)
Zolgensma® (Onasemnogene Abeparvovec-xioi)	0	10 (27.78) ^**a**^
Other	1 (1.54)	0
None	9 (13.84)	3 (11.11)
**Surgeries before, n (%)**^a^		
Spinal fusion	32 (49.23)	7 (19.44)
Spinal rods	24 (36.92)	8 (22.22)
Gastrostomy (G-tube)	9 (13.84)	11 (30.56)
Joint contracture	4 (6.15)	0
Tracheotomy	6 (9.23)	8 (22.22)
None	26 (40.00)	17 (47.22)
**Current tools/equipment use, n (%)**^a^		
Breathing machine/mechanical ventilation	15 (23.1)	11 (30.56)
Feeding tube	5 (7.7)	10 (27.78)
Suction machine to help clear throat	13 (20.0)	16 (44.44)
Cough assist machine	22 (33.8)	24 (66.67)
Walking frame	2 (3.1)	2 (5.55)
Wheelchair	53 (81.5)	20 (55.56)
Other	13 (20.0)	9 (25.00)
None	6 (9.23)	6 (16.67)

Mos., months; Yrs., years

^a^Answer options are not mutually exclusive of one another

**Fig. 2:**
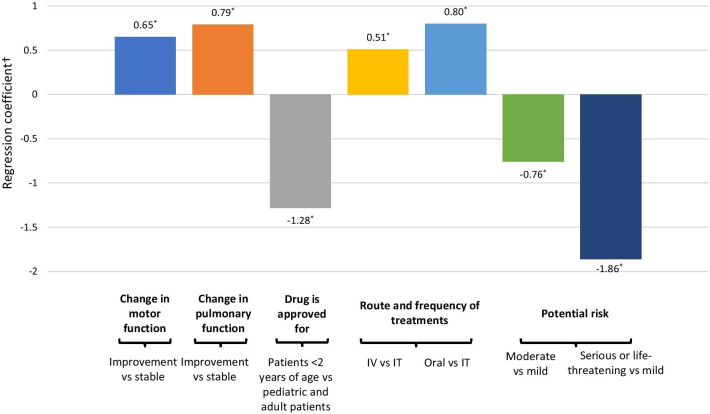
Preference for treatment attributes of individuals with SMA and their caregivers. *Results are statistically significant. ^†^Regression coefficient > 0 shows preference for a treatment attribute; regression coefficient < 0 shows aversion to a treatment attribute

Figure 2 and Appendix Table 4 present the conditional logit model estimates for the SMA treatment preferences. Overall, better improvement in motor and breathing function was highly valued (regression coefficient (RC): 0.65, 95% confidence interval [CI]: 0.47–0.83 and RC: 0.79, 95% CI: 0.60–0.98, respectively); and oral medication and one-time infusion were strongly preferred over repeated IT injections (RC: 0.80, 95% CI: 0.60–0.98 and RC: 0.51, 95% CI: 0.30–0.73, respectively). Patients least preferred an age-restricted label/approved use (≤ 2 years of age) and serious/life threatening risk (RC: − 1.28, 95% CI: − 1.47 to − 1.09 and RC: − 1.83, 95% CI: − 2.11 to − 1.60, respectively).

In the overall survey sample, when changing the route of admission from repeated IT injections to one-time IV injection, the results yield approximately 0.78 (0.51/0.65) times as much utility as increasing efficacy from stabilization to better improvement in motor function, signifying a preference for higher efficacy. On the other hand, changing route of admission from repeated IT injections to oral daily medication yields approximately 1.2 (0.80/0.65) times as much utility as increasing efficacy from stabilization to better improvement in motor function. All of the findings suggested a much greater preference weight was given to the oral daily administration, rather than IV injection. Likewise, reducing side effect severity from ‘moderate risk’ or ‘serious, life-threatening’ to ‘mild risk’ yields approximately 1.2 (0.76/0.65) and 2.9 (1.86/0.65) times as much utility, respectively, as increasing efficacy from stabilization to better improvement in motor function, signaling a stronger preference for risk minimization over additional efficacy gain.

The cross-attribute results related to breathing, such as preference on oral administration and risk aversion were similar to those of motor function. Changing the route of admission from repeated IT injections to one-time IV injection, the results yield approximately 0.65 (0.51/0.79) times as much utility as increasing efficacy from stabilization to better improvement in breathing function. Changing route of admission from repeated IT injections to oral daily medication yields approximately 1.0 (0.80/0.79) times as much utility as increasing efficacy from stabilization to better improvement in breathing function. Moreover, when looking at reducing potential side effect severity from ‘moderate risk’ or ‘serious, life-threatening’ to ‘mild risk’ yields approximately 0.96 (0.76/0.79) and 2.4 (1.86/0.79) times as much utility, respectively, as increasing efficacy from stabilization to better improvement in breathing function.

**Fig. 3:**
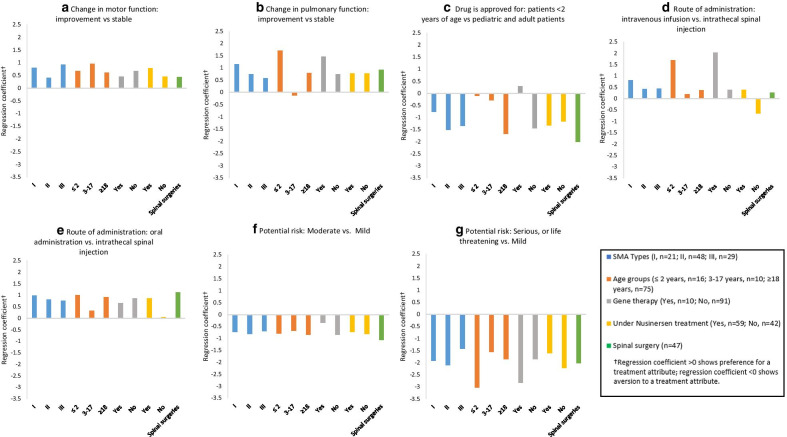
Conditional logit model estimates for SMA treatment preferences by sub-groups. **a** Change in motor function: improvement versus stable. **b** Change in pulmonary function: improvement versus stable. **c** Drug is approved for: patients < 2 years of age versus pediatric and adult patients. **d** Route of administration: intravenous infusion versus intrathecal spinal injection. **e** Route of administration: oral administration versus intrathecal spinal injection. **f** Potential risk: moderate versus mild. **g** Potential risk: serious, or life-threatening versus mild

Figure 3 and Appendix Table 5 present the conditional logit model estimates for SMA treatment preferences by multiple sub-groups: (1) Types of SMA—type 1, type 2, and type 3; (2) Age groups— ≤ 2 years, 3–17 years, and ≥ 18 years; (3) Gene Therapy—Yes/No, (4) nusinersen—Yes/No; (5) Spinal surgery—Yes/No. Regardless of subgroup, all groups preferred improvement in both motor and breathing function than stabilization of these functions, all else being equal. Improvement in breathing function is valued more than in motor function for patients with type 1 and 2 SMA whereas improvement in motor function is more important for patients with type 3 SMA. When evaluating who the drug is approved for, all subgroups preferred drugs that were approved for adults and children. However, this was no longer significant when examining the subgroups of children (e.g., age ≤ 2 years old, and 3–17 years) and those who were on gene therapy, as caregivers did not have a preference as to who the drug was approved for. When evaluating the route and the frequency of treatments, all subgroups preferred the oral daily medication. Interestingly, those who were not currently under nusinersen treatment, were less likely to prefer the one-time IV infusion rather than the IT spinal injection that is done 3–6 times per year (RC: − 0.65, *p* value < 0.05). A related point to consider is that a stronger preference for the oral daily medication over repeated IT spinal injection was found when examining the subgroup of patients who had previous spinal surgeries than in the overall sample. The preference for oral daily is independent of SMA treatments patients were receiving at the time of the survey (except for patients not receiving nusinersen treatment).

## Discussion

The treatment landscape for SMA has undergone tremendous changes with three FDA approved therapies becoming available in the last few years. It is therefore critically important to understand how patients and caregivers view treatment choices. This study is the first-ever discrete choice experiment to characterize decision-making and benefit-risk trade-offs for SMA treatment characteristics. These findings can inform the perceptions and preferences of SMA patients and their caregivers to stakeholders including regulators, patients, patient advocacy group such as CureSMA, and providers.

Across patient-centered treatment attributes to address the variety and severity of impairments and disabilities that accompany SMA, findings from this study identified the overall preferred treatment attributes including improvement in motor function, breathing function, oral administration, broad indication without age limits, and minimal risk. Overall, the preference on treatment characteristics were quite robust across demographic groups and clinical subgroups. Respondents’ age, sex, SMA type and associated characteristics (e.g., age of diagnosis ≤ 18 months), did not show a difference in direction of conditional logit estimates for patient preference.

Not surprisingly, improvement in motor and breathing function were highly valued over stabilization of each function, regardless of subgroup. For breathing function, the strength in preference for improvement over stabilization was the greatest among patients with the greatest severity of disease. One-time IV infusion and oral daily are preferred frequency of treatments vs. repeated IT administration across all SMA patients (β = 0.80). The preference is even stronger for oral daily among very young patients (β = 1.01), adult patients (β = 0.92), and patients with previous spinal surgeries (β = 1.12). Those not currently under an IT injection treatment, were less likely to prefer the one-time IV infusion rather than the IT spinal injection that is done 3–6 times per year (RC: − 0.65, *p* value < 0.05). This finding warrants further research.

The route of administration is an important treatment attribute because of the challenges of IT administration for SMA patients, particularly for those with severe scoliosis and spinal surgeries. The challenge of IT treatment may be further complicated by its treatment burden (travel, time from school/work) to receive IT injection among adults which may provide reasoning as to why oral route of administration was preferred. It is surprising that respondents expressed a stronger preference for oral daily administration than the one-time IV infusion except for those who were treated with gene replacement therapy. One possible explanation might be that although all treatment profiles are hypothetical in this experimental design, patients and caregivers would have naturally associated treatment attributes to actual approved treatments, as such one-time treatment may have suggested gene replacement therapy for some patients, which may have influenced treatment preference. Moreover, broad indication is strongly preferred for all patients except those ≤ 2 years of age, which currently have an effective gene replacement therapy available for this age group.

In using this DCE methodology, it is important to evaluate the preferences in the context of other features, examining the relative size of those preferences compared to other preferences. In doing so, the cross-attribute results found that for most patients (e.g., very young patients, adult patients, or patients with spinal surgeries), there may be willingness to trade off higher efficacy for less invasive, less risk, and more convenient route and frequency of treatment. When reducing side effect severity from ‘moderate risk’ to ‘mild risk’ the utility was 20% more than utility of moving from stabilization to better improvement in motor function. When reducing side effect severity from ‘serious, life-threatening’ to ‘mild risk’, this ratio was more than 5 times greater yielding 2.9 (1.86/0.65) times as much utility, as increasing efficacy from stabilization to improvement, suggesting a stronger preference for risk minimization over additional efficacy gain.

Likewise, the utility of changing from repeated IT injections to oral daily medication was 20% more than the utility of moving from stabilization to improvement; for adults, this ratio was about 50% more. These findings are consistent with previous research that has found stabilization in treatment to be highly meaningful [39], and although improvement in treatment is obviously preferred if given in comparison alone, patients would prefer to maintain stabilization if that means other treatment attributes would be prioritized (i.e. route of administration, side-effect severity, etc.). In a European SMA survey conducted by Rouault et al. (2017), the authors found that 96.5% of respondents believed that “stabilization of their current clinical state through treatment would represent progress” [39]. These findings demonstrate that SMA patients and caregivers deem stabilization as a very meaningful marker for their quality of life. This should be taken into consideration by clinicians and drug manufacturers, when developing new drugs and understanding benefits and risks associated with such treatments.

A previous study assessing patients’ perceptions of benefit-risk decision-making for SMA therapies found no strong correlations between risk tolerance and patient/disease attributes [40]. However, this was before the approval of any therapeutic options. Now that new treatment options are available to patients, the benefit-risk decision making we observed in this study found that patients had a lower willingness for high risk therapies even if the benefit trade-off was higher.

Evaluating patient preferences in our study should be interpreted in the context of two innovative treatments used by some of the study respondents as two important subgroups (i.e. onasemnogene abeparvovec-xioi and nusinersen), although the use of gene replacement therapy in our study only represents a small sample size. A comparison on the conditional logit estimates found that the preferences between nusinersen users and non-users were quite similar, except for the strong preference for oral daily administration among those receiving nusinersen (repeated IT spinal injection). The Pacione et al. (2019) qualitative study on the perspectives of nusinersen treatment, characterized the patients/caregivers with SMA who did not want or were unsure about nusinersen and found that their decision about pursuing nusinersen treatment was quite nuanced, challenging and context-specific [41].

It is important to note that all treatments presented to patients and caregivers in this study are hypothetical and were automatically generated by computers. As such, one is supposed to not associate the preference (or lack of) for certain treatment attributes to actually approved treatments and treatments currently being developed. We recognized that it may be inevitable that a distinct treatment attribute may be linked to a specific treatment and/or patient experience with such a treatment, which may have influenced treatment choices. For example, the actual 10 gene-replacement therapy users did not appear to prefer the oral daily administration, and instead had a strong preference for the one-time IV administration, which may suggest the experience of one-time IV gene transfer is well tolerated and could be appealing for patients who are eligible.

With new treatments potentially turning a life-threatening disease into a chronic disease, clinicians may need to conform to a patient-centered care model that prioritizes a patient’s/family’s values, goals, and knowledge as well as patient’s self-awareness. The attention to the values and priorities from clinicians may create a shared decision making between patients and providers.

There were several limitations in the study. First, the survey sample was drawn from a SMA patient organization membership database, representing a highly informed and engaged patient population, and therefore the results may not represent all SMA patients in the United States. In particular, our sample only had one patient with type 4 SMA. Second, SMA type, motor function, and treatment status were self- or caregiver-reported. No attempts were undertaken to verify such information. Third, although affordability was a concern for many patients and caregivers, patient out-of-pocket cost was not included in the treatment profile due to administration burden and sample size limitation. Furthermore, a number of factors beyond the efficacy and side effects of treatment may influence perception and preferences on treatment characteristics among patients with SMA including disease knowledge, transportation needs, and functional status as well as insurance approval or financial assistance for very expensive SMA treatments. These factors may also interact with each other with large scope of complexity and difficulty involved in the treatment decisions for patients with SMA.

In regard to availability of actual treatments on the market, preference of one attribute may be confounded by related attributes based on actual, approved SMA treatments on the market (e.g. association of one-time infusion with gene therapy). Additionally, treatments were not explicitly described during the interview process in terms of scientific facts of effects and adverse effects of these treatments. Information about the purpose of the survey and directions for completing the survey were provided but information about specific SMA treatments and their effects were not provided. Patients only made responses based on their own personal knowledge, which limited the chance of interviewer bias. This is because the DCE study design was hypothetical and the purpose of this study was not to explicitly describe SMA treatments but rather common treatment attributes and characteristics. Thus, allowing us to understand a patient’s desired treatment in a hypothetical scenario to provide insight as to whether patients would be interested or prefer an option over the others if it was available.

Lastly, the hypothetical treatment attributes and levels may over-simplify any treatment in the real world; treatment decision has to be made in clinical context and tailored to patient specific needs. Nonetheless, these hypothetical scenarios provide insight into patient and caregiver preferences that can inform future drug development and drive the implications for future research of this highly rare, complicated, and expensive disease.

## Conclusion

Improvement in motor function, breathing function, broad indication, oral daily administration, and minimal risk are preferred treatment attributes among all SMA patients. Cross-attributes trade-off decision suggested a lower willingness for a high-risk therapy despite additional efficacy gain. For some patients, there may be willingness to trade off additional gains in efficacy for a change in route of administration from repeated IT administration to oral medication. Patient preference and clinical context should be an integral part of treatment decision process and tailored to individual patient needs.

## Data Availability

The datasets generated and/or analyzed during the current study are not publicly available due to patient confidentiality and the consent agreement but are available from the corresponding author on reasonable request.

## Appendix

See Tables 4 and 5 below.

**Table 4:** Attribute preferences for SMA treatment

	Coefficient	SE	95% CI		*p* value
Level of change in motor function (ref: Stable)					
Better	0.65	0.09	0.47	0.83	0.000
Change in breathing function (ref: Stable)					
Better	0.79	0.10	0.60	0.98	0.000
Drug is approved for: (ref: Pediatric and adult patients with SMA)					
Pediatric patients with SMA under 2 years of age	− 1.28	0.10	− 1.47	− 1.09	0.000
Route and frequency of treatments (ref: Intrathecal spinal injection—3–6 times per year)					
Intravenous injection—one-time treatment	0.51	0.11	0.30	0.73	0.000
Oral—daily medication	0.80	0.13	0.55	1.04	0.000
Treatments potential risk (ref: Mild risk)					
Moderate risk	− 0.76	0.13	− 1.01	− 0.50	0.000
Serious, or life-threatening risk	− 1.86	0.13	− 2.11	− 1.60	0.000

SE, standard error; CI, confidence interval

**Table 5:** Preferences for SMA treatment by sub-groups

	SMA types			Age groups			Currently under gene therapy		Currently under Spinraza® (nusinersen) treatment	
	Type I (n = 21)	Type II (n = 48)	Type III (n = 29)	≤ 2 years (n = 16)	3–17 years (n = 10)	≥ 18 years (n = 75)	Yes (n = 10)	No (n = 91)	Yes (n = 59)	No (n = 42)
Level of change in motor function (ref: stable)										
Better	0.80*	0.41*	0.92*	0.68*	0.95*	0.61*	0.46	0.67*	0.79*	0.45*
Change in breathing function (ref: stable)										
Better	1.16*	0.75*	0.58*	1.71*	− 0.12	0.80*	1.47*	0.75*	0.77*	0.78*
Drug is approved for: (ref: pediatric and adult patients with SMA)										
Pediatric patients with SMA under 2 years of age	− 0.76*	− 1.51*	− 1.35*	− 0.10	− 0.28	− 1.68*	0.30	− 1.44*	− 1.33*	− 1.16*
Route and frequency of treatments (ref: intrathecal spinal injection—3–6 times per year)										
Intravenous injection—one-time treatment	0.82*	0.42*	0.44*	1.69*	0.19	0.38*	2.03*	0.39*	0.39*	− 0.65*
Oral—daily medication	0.98*	0.82*	0.76*	1.01*	0.33	0.92*	0.65	0.87*	0.87*	0.05
Treatments potential risk (ref: mild risk)										
Moderate risk	− 0.73*	− 0.81*	− 0.70*	− 0.79*	− 0.68	− 0.84*	− 0.34	− 0.84*	− 0.73*	− 0.81*
Serious, or life-threatening risk	− 1.91*	− 2.11*	− 1.43*	− 3.03*	− 1.55*	− 1.85*	− 2.83*	− 1.85*	− 1.61*	− 2.22*

^*^*p* < 0.05; ***p* < 0.01; ****p* < 0.001

## Abbreviations

DCE: Discrete choice experiment
FDA: Food and Drug Administration
IT: Intrathecal
IV: Intravenous
RC: Regression coefficient
SMA: Spinal muscular atrophy
SMN: Survival motor neuron
SMN2: Survival motor neuron-2 (SMN2)
